# Molecular Mechanism for the Selective Presentation of Antigenic Peptides by Major Histocompatibility Complex Class I and Class II Molecules: A Hypothesis

**DOI:** 10.3390/cimb47110945

**Published:** 2025-11-13

**Authors:** Bao Ting Zhu

**Affiliations:** Shenzhen Key Laboratory of Steroid Drug Discovery and Development, School of Medicine, The Chinese University of Hong Kong, Shenzhen 518172, China; BTZhu@CUHK.edu.cn

**Keywords:** antigen presentation, major histocompatibility complex molecules, T cell receptors, antigen-presenting cells, dendritic cells, extracellular vesicles

## Abstract

The major histocompatibility complex (MHC) class I and class II molecules (abbreviated as MHC-I and MHC-II, respectively) are specialized in antigen presentation. Unlike the T cell receptors (TCRs), which have great variability, the MHC-I and MHC-II molecules essentially have no variability at all. It is apparent that the MHC-I and MHC-II molecules per se do not have the built-in ability to distinguish the huge populations of self-peptides from antigenic non-self-peptides. At present, the precise mechanism underlying the selective presentation of antigenic peptides by both MHC-I and MHC-II molecules is unclear. For an MHC-II molecule to gain the ability to selectively present antigenic (mostly foreign) peptides, it is hypothesized herein that all naïve CD4^+^ T cells in the body will release extracellular vesicles (EVs), which are specially designed for antigen-presenting cells (APCs); these EVs contain mRNAs that will be delivered to APCs and translated into an intracellular version of the TCR proteins (iTCR^II^), which will help select antigenic peptides for presentation by the MHC-II molecules. Similarly, it is hypothesized that the fully activated CD4^+^ T cells will also release EVs, and these EVs contain different mRNAs for another intracellular version of the TCR proteins (iTCR^I^), which will help pathogen-infected somatic cells to select the antigenic peptides (mostly from invading pathogens) for presentation by the MHC-I molecules. Understandably, while the iTCR^II^ proteins will work closely with the MHC-II molecules in the exogenous endocytic pathway, the iTCR^I^ proteins will work closely with the MHC-I molecules in the endogenous pathway. In this paper, a few other related hypotheses are also proposed, which jointly offer a plausible mechanistic explanation for the selective presentation of antigenic peptides by both MHC-I and MHC-II molecules. While the proposed hypotheses are partly supported by some experimental observations, it is hoped that these hypotheses will promote discussion and experimental testing of the mechanisms underlying the complex process of selective antigen presentation.

## 1. Introduction

While the functions of B cells and antibodies are mostly for ridding the body of soluble/free toxins, viruses and bacteria [1], the functions of T cells are for monitoring the status of host cells for signs of viral infection and malignant transformation [2,3]. This difference in the functions of B and T cells is also distinctly reflected in how B and T cells recognize their respective antigens. While antibodies can directly bind to antigens in solution or present on the surface of pathogens [1,2], the majority of T cells are specialized in recognizing antigens only after they have been processed by the host cells and presented, in the form of short peptides, in complex with a plasma membrane-bound major histocompatibility complex (MHC) class I or class II molecule (MHC-I or MHC-II) [2,3,4,5,6,7,8].

MHC-II molecules are expressed primarily in cells of the immune system, such as dendritic cells (DCs), macrophages, B cells, and thymic cortical epithelial cells [9,10,11]. These cells are generally considered as the professional antigen-presenting cells (APCs). In addition, many somatic cells can also be induced to express MHC-II molecules under certain conditions, such as during sustained inflammation and under interferon-*γ* (IFN-*γ*) stimulation [11]. In comparison, the MHC-I molecules are expressed in most nucleated somatic cells, and their presence is crucial for cytolysis of pathogen-infected somatic cells by CD8^+^ cytotoxic T cells. These CD8^+^ T cells kill cells on which they recognize the antigen and help eliminate cells infected with viruses or bacteria which live in their cytosolic compartments. In addition, CD8^+^ T cells also recognize mutated self-proteins on cancer cells and help eliminate them, or recognize allogeneic MHC-I molecules expressed on organ transplants and cause their rejection [6,12]. Both naïve CD4^+^ and CD8^+^ T cells can become armed effector T cells after encountering their cognate antigens once they have been processed and presented by the activated DCs.

It is known that immunoglobulins (Igs), B cell receptors (BCRs) and T cell receptors (TCRs) all have great variability (estimated total numbers > 10^12^). Their diversity results from the built-in mechanisms of the body’s immune system, such as the combinatorial diversity of multiple germline gene fragments, the *V(D)J* junction rearrangement diversity, junctional flexibility, *N*-region nucleotide insertion, and combinatorial diversity of the multiple chains [2]. Unlike Igs, BCRs and TCRs, however, the MHC-I and MHC-II molecules essentially have no variability at all. While several hundred different allelic variants of MHC-I and MHC-II molecules have been identified in humans, any one individual only expresses a very small subset of these molecules—up to 6 different MHC-I molecules and about 12 different MHC-II molecules [9,13,14,15]. Structural analysis revealed that the MHC-I molecules can hold any peptides which have the right size (8–10 amino acids in length, 9 preferred) and meet the general structural requirements during their presentation to cytotoxic CD8^+^ T cells [16,17,18]. Similarly, the MHC-II molecules in APCs can hold any antigenic peptides with a suitable size of 13–18 amino acids in length during their presentation to naïve CD4^+^ T cells [11,16]. It is apparent that the MHC-I and MHC-II molecules per se do not have the built-in ability to distinguish the huge number of self-peptides from foreign antigenic peptides. At present, the precise mechanism by which MHC-I and MHC-II molecules selectively present antigenic non-self-peptides is not understood. In this paper, a number of related hypotheses are proposed, which offer a tentative mechanistic explanation for the selective presentation of antigenic non-self-peptides by both MHC-I and MHC-II molecules. The proposed hypotheses are briefly discussed below, along with a discussion of the rationale and the supporting evidence which is mostly scattered in the literature in bits and pieces.

## 2. Hypotheses and Discussion of the Supporting Evidence

### 2.1. Many APCs Ectopically Express an Intracellular Version of the TCR Proteins Originally Created in Naïve CD4^+^ T Cells

As mentioned above, the MHC-II molecules in an APC can hold different peptides with the right size and suitable general structure, and as such, these proteins do not have the built-in ability to distinguish self-peptides from foreign antigenic peptides. To gain the ability to selectively present only antigenic peptides, it is hypothesized that all naïve CD4^+^ T cells in the body will release extracellular vesicles (EVs), which are specially designed for APCs, i.e., these EVs will selectively deliver “cargos” for the APCs. These naïve CD4^+^ T cell-produced EVs contain mRNAs that can be translated into an intracellular version of the TCR proteins in APCs, and these intracellular TCRs have the same antigen-binding site (binding pocket) as the regular TCRs present on the surface of a naïve CD4^+^ T cell that produces these EVs. Since one naïve CD4^+^ T cell can only produce one type of TCRs, the EVs released from a naïve CD4^+^ T cell will also contain the mRNAs for a single type of intracellular TCR proteins. Notably, these naïve CD4^+^ T cell-produced mRNAs will only be translated into an intracellular version of the TCR proteins in APCs in specified subcellular compartments (but not on their cell surface). Moreover, these intracellular TCRs will work jointly with the MHC-II molecules (most likely in the late endosomes of the endocytic pathway) [19], and their function is to select antigenic (mostly foreign) peptides for presentation by the APC’s MHC-II molecules. For ease of description, this ectopically expressed intracellular TCR is referred to as **iTCR^II^** (here, “**i**” denotes its intracellular localization, and the superscript “**II**” denotes its functional coupling with the MHC-II molecule for the selective presentation of antigenic peptides). It should be noted that these ectopically expressed iTCR^II^ proteins will not be used for selecting and presenting antigenic peptides to the MHC-I molecules; a slightly different mechanism is used for MHC-I molecules (discussed later).

Regarding the mechanism by which the naïve T cell-derived EVs can identify APCs to deliver their cargos (including mRNAs), it is speculated that a set of transmembrane proteins are selectively present on the surfaces of EVs and APCs, and these proteins will serve as high-affinity binding partners (just like receptors and ligands). The selective binding interactions between these binding partners will dictate high-affinity binding interactions between the EVs and APCs, and with the help of other protein machineries specialized in membrane fusion, the outer membrane of an EV will fuse with the plasma membrane of an APC, resulting in the delivery of the luminal cargos of an EV to the APC. The proposed mechanism is supported by earlier studies showing that the outer membrane of an EV can readily fuse with the plasma membrane of a target cell, which is followed by the discharge of the luminal cargos (containing proteins, RNAs and other components) into the cytoplasm of the target cell [19]. Additionally, it is also suggested that there is a mechanism by which the cargos carried by the EVs can be delivered to the right subcellular compartments of the APCs, but not ended up in the endolysosomal compartments as usually seen following endocytosis of the EV particles.

Since there are so many different naïve CD4^+^ T cells circulating in the body and each one carries a different TCR (i.e., with a different binding site structure), it is speculated that an APC can receive EVs (which contain the iTCR^II^ mRANs) from many different naïve CD4^+^ T cells. In other words, the iTCR^II^ mRNAs from many different naïve CD4^+^ T cells may be concomitantly translated into different clones of iTCR^II^ proteins inside an APC as a way to increase efficiency. This suggestion is highly possible based on the fact that a vast majority of naïve CD4^+^ T cells (and their TCRs) eventually will not be activated by any antigens (because their TCRs cannot bind any antigens). If each APC is only permitted to receive EVs from one naïve CD4^+^ T cell, then the outcome is that most APCs in the body eventually are incapable of presenting antigens to activate naïve CD4^+^ T cells. Alternatively, if a newly formed APC can receive EVs (and their cargos) from many different naïve CD4^+^ T cells, it will beneficially increase the chances for an APC to present antigenic peptides. However, it is also believed that an APC will not be allowed to continuously accept EVs (and their cargos) from many other naïve CD4^+^ T cells forever. It is speculated that once an APC starts to present an antigen (which means that one of the ectopically expressed iTCR^II^s can selectively bind and select an antigenic peptide), then this APC will cease to receive additional EVs from other naïve CD4^+^ T cells. As a result of this mechanism, an APC in the body usually will only be allowed to selectively present one clone of the same antigens. In so doing, the APC will have the ability to selectively inform the naïve CD4^+^ T cell (which is being activated by the APC through antigen presentation) the origin of the antigen (i.e., whether the antigenic peptide is derived from a virus or a bacteria, or from a cellular component, etc.), through distinct combinations of various activation signals (more discussion is provided later). Alternatively, if multiple antigenic peptides (e.g., one from the engulfed virus and one from a mutated cellular protein) are concomitantly presented by an APC, it would be impossible for the APC to selectively inform different naïve CD4^+^ T cells where each antigenic peptide comes from.

It is known that during antigen processing in an APC, the antigenic peptides may come from different subcellular compartments. These compartments, which are usually separated by cellular membranes, include the cytosol and various vesicular compartments involved in endocytosis and secretion. Pathogenic bacteria that proliferate outside the cells can be taken up, along with their toxic products, by phagocytosis, receptor-mediated endocytosis, or macropinocytosis into endosomes and lysosomes. Similarly, virus particles and parasite antigens in extracellular fluids can also be taken up by these routes and degraded. In professional APCs, the late endosomes contain selectively expressed protein-degrading enzymes that preferentially catalyze the formation of 13–18 amino acid-long peptides suited for MHC-II binding and presentation. Additionally, these professional APCs also have a unique form of late endosomes, i.e., the MHC-II-containing compartment, in which final protein degradation and peptide loading into the MHC-II molecules take place [20].

At present, the exact mechanism by which an antigenic peptide is picked for loading into the binding groove of an MHC-II molecule is still not clearly understood, and a tentative model is entertained here. Structurally, it is known that an MHC-II molecule contains two membrane-bound *α* and *β* chains of similar size, and they associate through noncovalent interactions [2,5]. After the MHC-II is synthesized in the rough ER (RER), its peptide-binding groove is associated with a protein called the invariant chain (Ii or CD74) which blocks the binding of any endogenously derived peptides while still in the RER [21,22]. As the MHC-II–invariant chain complex moves through the endosomal compartment, the invariant chain is gradually degraded. However, a short fragment of the invariant chain, termed CLIP, remains bound to MHC-II, in a similar way as an antigenic peptide [21,22]. It is generally believed that CLIP physically occupies the peptide-binding groove of the MHC-II, which prevents any premature binding of other processed peptides.

To catalyze the exchange of CLIP with an antigenic peptide, HLA-DM (a nonclassical MHC-II molecule) is required [23,24,25]. HLA-DM has a similar structure as MHC-II, composed of a heterodimer of *α* and *β* chains, but it does not have the peptide-binding groove [23,25,26]. Studies have shown that HLA-DM can associate with the *β* chain of MHC-II and performs the function of removing CLIP in MHC-II’s binding groove [25,26]. Structural analysis has revealed that the P1 pocket in the peptide-binding groove is a major determinant of peptide binding affinity [2,25,26]. When HLA-DM is bound tightly to MHC-II, the binding of a peptide into the binding groove of MHC-II becomes sterically blocked in the P1 pocket, and the presence of HLA-DM results in conformational changes unfavorable to peptide binding [25,26]. This is the structural basis for the removal of CLIP from the peptide-binding groove, and it is also the reason for the very low binding affinity of other peptide substrates in the binding groove when HLA-DM is still bound to the MHC-II molecule. After CLIP is removed from the binding groove of the MHC-II molecule, HLA-DM will bind tightly to the empty MHC-II molecule to help stabilize its structure and also to keep its peptide-binding groove open. Here, it is speculated that if iTCR^II^ protein recognizes a peptide that is transiently and loosely bound to an MHC-II molecule, then the loading of the peptide to MHC-II’s binding groove will be confirmed with the formation of the HLA-DM–MHC-II–peptide–iTCR^II^ complex. An alternative possibility is that the iTCR^II^ may bind the antigenic peptide first to form an initial complex, and then the iTCR^II^ presents the selected peptide to the HLA-DM–MHC-II complex. In either case, it is further speculated that the high-affinity binding of the peptide jointly with both iTCR^II^ and MHC-II will cause HLA-DM to dissociate from MHC-II such that the MHC-II molecule can fully receive the peptide for loading, and in the meantime, the dissociated HLA-DM will, in turn, cause iTCR^II^ to dissociate from that complex, likely through the HLA-DM–iTCR^I^ interactions.

In the literature, it is widely held that HLA-DM has the so-called “editing function” of the peptides being loaded into the binding groove of MHC-II molecules [23,24,25,26]. Based on the above explanation, it appears that iTCR^II^ actually determines which antigenic peptide will be selected for MHC-II loading. In fact, HLA-DM is not directly involved in selecting (or “editing”) the peptide but simply serving as a non-specific helper in this selection process. After the antigenic peptide is bound to the binding groove of an MHC-II molecule, the iTCR^II^ will remain inside the late endosomes, where it will take part in the next round of antigen selection and presentation to another MHC-II molecule.

According to the proposed hypothesis, most APCs have the ability to selectively fuse with the EVs released by naïve CD4^+^ T cells to receive the iTCR^II^ mRNAs; otherwise, these APCs cannot selectively present antigenic peptides in complex with the MHC-II molecules. While DCs, macrophages and B cells are generally considered as professional APCs specialized in antigen presentation, most somatic cells in the body usually do not have the APC-like ability to present antigens for the selective activation of naïve CD4^+^ T cells. However, under certain conditions, some of the pathogen-infected somatic cells may transiently acquire the function to present antigens like professional APCs such that the body can more efficiently present those antigens derived from the invading pathogens for activation of naïve CD4^+^ T cells. Under such conditions, one of the most important steps to arm these regular somatic cells to be APC-like cells is that these somatic cells need to selectively acquire iTCR^II^ mRNAs produced by naïve CD4^+^ T cells just like the APCs do. The presence of the iTCR^II^ proteins in these infected somatic cells will then enable them to selectively present antigenic peptides from the invading pathogens. In addition to acquiring the iTCR^II^ mRNAs, these nonprofessional APCs also need to express MHC-II molecules and relevant costimulatory signals. After all these steps, they are properly deputized for antigen presentation, usually for a given duration under certain situations.

Among the various professional APCs, it is known that there are marked differences in the levels of MHC-II expression. In most cases, MHC-II expression depends on the levels of their activation. APC activation usually occurs following their interaction with pathogens (containing pathogen-associated molecular patterns, PAMPs) via the pattern recognition receptors (PRRs) coupled with cytokine signaling, which jointly alters gene expression in these APCs, including increased expression of MHC-II molecules.

B cells usually constitutively express MHC-II molecules at low levels, and possess antigen-specific surface receptors [11]. Theoretically, B cells are excellent professional APCs, as these cells can recognize and selectively concentrate the invading pathogens facilitated by the specific BCRs present on the surface of these cells. It is speculated that B cells (like other professional APCs) also express iTCR^II^s (through receiving the EVs released from CD4^+^ naïve T cells). Assume that a B cell internalizes extracellular protein particles from a pathogen through its BCR-mediated endocytosis, and the internalized proteins contain the antigenic peptides that have high affinity for the iTCR^II^s and then selected for presentation on its surface in complex with the MHC-II molecules. These B cells will present the antigenic peptides to a naïve CD4^+^ T cell for its activation. The activated CD4^+^ T cell will subsequently be differentiated into a special clone of helper T cells, which can selectively activate this clone of B cells, leading to its proliferation and clonal expansion. Consequently, more B cells with the same ability to recognize the invading pathogens will be present in the circulation to capture the pathogens (or their proteins) for antigen processing and presentation. In addition, the specific antibodies produced and released by this clone of B cells (i.e., plasma cells) will also help enhance the phagocytosis of the invading pathogens by other professional DCs or enhance their clearance by macrophages [1]. Notably, when the DCs capture these “antibody-tagged” pathogens, some of the DCs may also have the ability to selectively present pathogen-derived peptides as these DCs may already have expressed the same cognate iTCR^II^ proteins.

**Figure 1:**
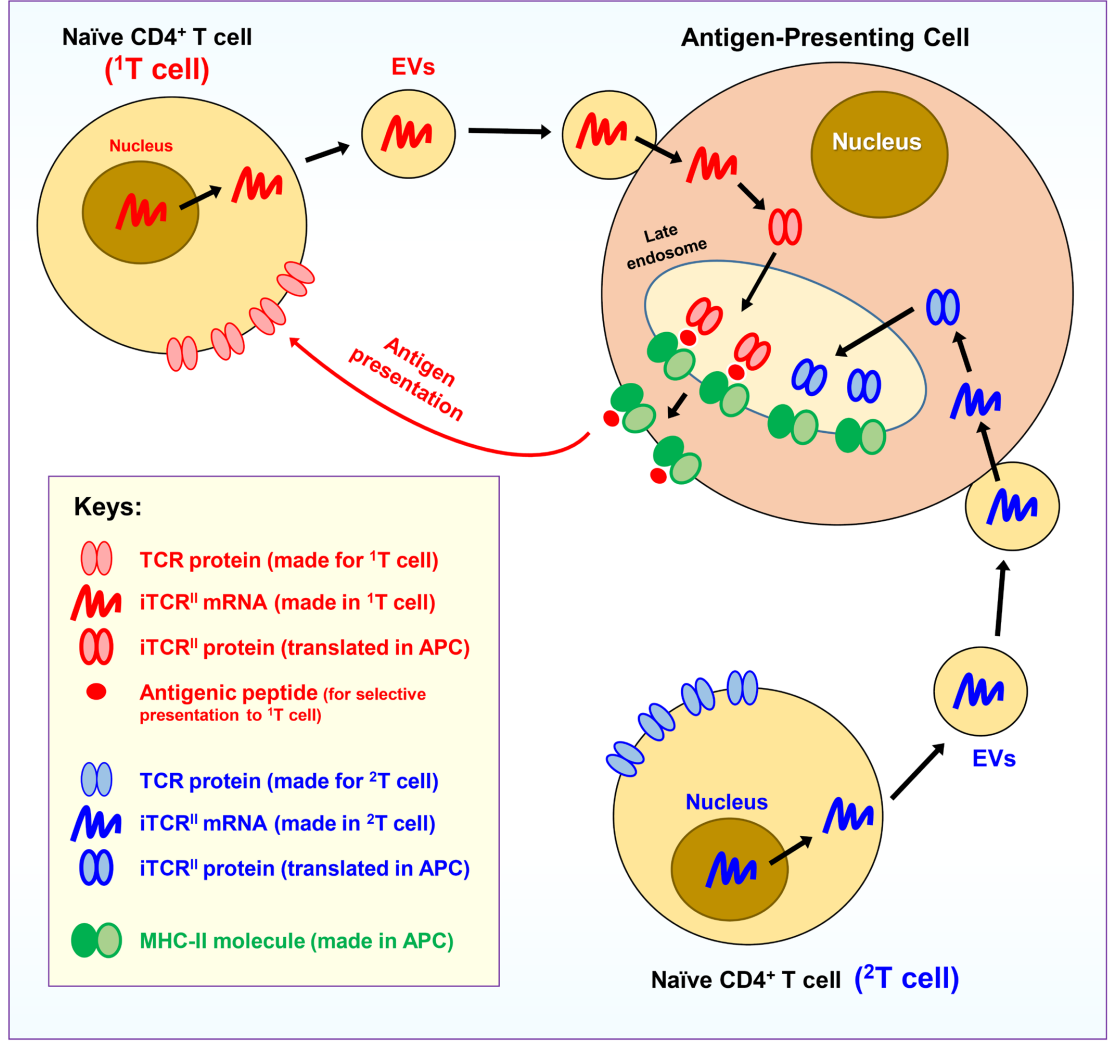
**Hypothesis I. Antigen-presenting cells (APCs) can ectopically express an intracellular version of the TCR proteins.** As depicted, it is hypothesized that all naïve CD4^+^ T cells in the body can release extracellular vesicles (EVs), which are specially designed to deliver “cargos” for APCs. The EVs contain mRNAs for an intracellular version of the TCR proteins (namely, **iTCR^II^**), which have the same antigen-binding site structure as the original version of the TCRs present on the surface of a naïve CD4^+^ T cell that produces the EVs. These iTCR^II^ proteins will be expressed in an APC in its late endosomes and will help to select antigenic peptides for presentation by the APC’s MHC-II molecules. As depicted, while an APC may receive mRNAs derived from many different naïve CD4^+^ T cells (in the figure, two representative T cells, i.e., **^1^T** and **^2^T**, are drawn), usually only a single type of antigens will be presented by an APC, and as a result, only one clone of the naïve CD4^+^ T cells (in this case, the **^1^T** clone) will be activated by an APC.

In summary, as depicted in Figure 1, it is hypothesized that all naïve CD4^+^ T cells in the body can release EVs to deliver iTCR^II^ mRNAs for APCs. These iTCR^II^ proteins have the same antigen-binding site structures as the original TCRs present on the surface of these naïve CD4^+^ T cells. These iTCR^II^ proteins will work in late endosomes to help select antigenic peptides for presentation by the MHC-II molecules. While an APC can receive mRNAs from many different naïve CD4^+^ T cells, it is believed that an APC normally will only present one single type of the antigenic peptides and thus will only activate one clone of the naïve CD4^+^ T cells.

Here, it should be noted that since all TCRs have already gone through the selection process during thymocyte maturation in the thymus, theoretically the TCRs can only bind antigenic (mostly foreign) peptides but not non-antigenic self-peptides. Because the peptide-binding site structure (and its amino acid sequence) of the ectopically expressed iTCR^II^ proteins in an APC is the same as the cognate TCRs present on the surface of the corresponding naïve CD4^+^ T cells, it is, therefore, not difficult to understand that the iTCR^II^ proteins in an APC normally will not bind self-peptides. Because of this property, most non-antigenic self-peptides normally are not presented on the surface of an APC in complex with its MHC-II molecules. In other words, APCs only selectively present antigenic peptides derived from engulfed pathogens or transformed cells.

### 2.2. An Activated Naïve CD4^+^ T Cell Has Multiple Fates

The engagement of a naïve CD4^+^ T cell with an APC usually takes place in the T-cell zone of a secondary lymphoid tissue. It is known that the high-affinity binding of the TCR of a naïve CD4^+^ T cell with the MHC-II–peptide complex on an APC, even aided with the binding of CD4 (jointly referred to as “Signal-1”), is not sufficient to fully activate a naïve T cell [2,27]. For the full activation to occur, the CD28 coreceptor on a CD4^+^ naïve T cell must also engage its ligand (CD80 or CD86) on the APC (which forms “Signal-2”) [2,27]. It is believed that the requirement of CD28 to engage its ligand on an APC serves as a safeguard which helps a naïve CD4^+^ T cell to reduce the likelihood that the peptide complexed to MHC-II of an APC is from the self-components. It is known that CD80 and CD86 (both are ligands of CD28) are present only on professional APCs such as DCs, macrophages, and activated B cells. Moreover, the levels of their expression are selectively increased following antigen uptake mediated by APC’s innate immune receptors (such as BCRs and PRRs). As such, the CD4^+^ naïve T cells, through specific interaction between its CD28 and APC’s CD80/CD86, will be assured that the peptide-presenting APC have just engulfed foreign antigens. On the other hand, if a peptide-presenting APC does not express high levels of CD80 or CD86, then a CD4^+^ naïve T cell will be alerted that the APC might be accidentally presenting a self-peptide. Lastly, an APC following the activation of its innate immune receptors will also be induced to secrete cytokines. Different cytokine mixtures are secreted depending on the types of APCs, the identities of the PRRs being activated, and the cytokine environments the APCs are in. The cytokines secreted by an APC (which forms “Signal-3”) are needed to complete the initial activation of a naïve CD4^+^ T cell [2,27]. Here, it is of note that in addition to the above-mentioned CD80/CD86–CD28 ligand–coreceptor partners (“Signal-2”), there are also other ligand–coreceptor partners, which are functionally similar but may help specify a different immunological context in which a naïve CD4^+^ T cell is activated.

Here, it is further hypothesized that following the initial activation of a naïve CD4^+^ T cell by an APC (usually in the T cell zone of a secondary lymphoid tissue), this CD4^+^ T cell will need to move back to the medulla regions of the thymus to undergo another round of negative selection. This step serves as a double check to ensure that this activated CD4^+^ T cell does not, by any chance, react strongly with any of the self-peptides presented by the medullary thymic epithelial cells (mTECs). If the activated CD4^+^ T cell does not bind to any of the self-peptides in the thymus with a meaningful binding avidity, which would mean that it has passed the double check, then this activated CD4^+^ T cell is licensed to undergo proliferation inside the thymus to become a small clone of CD4^+^ effector T cells. However, occasionally it may occur that some of the CD4^+^ T cells are found to have certain degrees of cross-reactivity against mTEC-presented self-antigens. As outlined below, it is hypothesized that these different CD4^+^ T cells will be allowed to differentiate inside the thymus into different subsets of immune cells with very diverse (even opposing) functions for regulating the body’s immune responses.

- A subset of the fully activated CD4^+^ T cells will become a unique group of effector/helper T cells which will release EVs specially designed to arm pathogen-infected somatic cells in the body for recognition by the cytotoxic CD8^+^ T cells (discussed in Section 2.3).
- A subset of the fully activated CD4^+^ T cells will become many other types of helper T cells which will take part in the fight against the invading pathogens (discussed in Section 2.4).
- Some of the fully activated CD4^+^ T cells will be transformed into naïve CD8^+^ T cells while they are still in the thymus (discussed in Section 2.5). It is speculated that all clones of naïve CD8^+^ T cells are formed in this way in the body, i.e., they all come from activated CD4^+^ T cells.
- If a fully activated CD4^+^ T cell happens to cross-react with self-peptides presented on mTECs (in complex with the MHC-II molecules), then this self-reactive CD4^+^ T cell will be converted into a clone of CD4^+^ regulatory T (T_REG_) cells, which will help suppress autoimmunity in the body (discussed in Section 2.6). On the other hand, if a CD4^+^ T cell is only partially activated by an APC, i.e., in the presence of Signal-1 but in the absence of Signal-2, it would mean that the antigenic peptide presented by the APC may not come from an invading pathogen. There are two plausible outcomes: If the partially activated CD4^+^ T cell is self-reactive against mTECs, then it will be differentiated into a subset of CD4^+^ regulatory T cells; however, if the partially activated CD4^+^ T cell has no self-reactivity against mTECs, then it will be transformed into a subset of CD4^+^ helper T cells, which might be involved in regulating anticancer immunity or autoimmunity (discussed in Section 2.6).
- Similar to a CD4^+^ T cell, if a CD8^+^ T cell (while still in the thymus) happens to cross-react with self-peptides presented on mTECs (in complex with the MHC-I molecules), then this self-reactive CD8^+^ T cell will be converted into a clone of CD8^+^ T_REG_ cells and involved in suppressing autoimmunity or anticancer immunity (discussed in Section 2.7).

### 2.3. A Subset of the Activated Effector CD4^+^ T Cells Will Release EVs Specially Designed to Arm Pathogen-Invaded Somatic Cells for Antigen Presentation

After a fully activated CD4^+^ T cell has successfully gone through another round of negative selection in the thymus, it will return to the T-cell zone of a secondary lymphoid tissue. The transcription of genes for both IL-2 and the α chain (CD25) of the high-affinity IL-2 receptor will be upregulated in this activated T cell. IL-2, along with other factors, will act on the activated CD4^+^ T cell, inducing robust cell division. This clone of activated CD4^+^ T cells will then differentiate into different subsets of effector/helper T cells, with each subset playing a specific role in the concerted immune responses against the invading pathogens.

Specifically, here it is hypothesized that a subset of the activated effector/helper CD4^+^ T cells will start to release EVs which specifically target recently infected somatic cells in the body, and these EVs contain mRNAs which can be translated into another intracellular version of the same TCR. Note that this version of the intracellular TCR will be expressed in these recently infected somatic cells (which will also start to express the MHC-I molecules), and its function is to work jointly with the MHC-I molecule to select antigenic peptides for presentation by MHC-I. For convenience, this intracellular version of the TCR protein is referred to as **iTCR^I^** (“**i**” denotes its intracellular localization, and the superscript “**I**” denotes its functional coupling with the MHC-I molecule). The iTCR^I^ molecules will be closely associated with cellular organelles of the internal protein metabolic pathway, in a similar manner as the MHC-I proteins.

Regarding the possible mechanism by which the iTCR^I^ protein selects an antigenic peptide and then presents it to an MHC-I molecule, it is speculated that iTCR^I^ may work closely with TAP and the peptide-loading complex (PLC) to select and then transfer the antigenic peptide to an MHC-I molecule. It is known that the peptide-binding sites of the MHC-I molecules are formed inside the lumen of RER, and, in fact, the MHC-I molecules are never exposed to the cytosol [2]. As a result, the peptide-binding pocket of MHC-I is actually empty while it is inside the RER lumen; this situation is unlike an MHC-II molecule which is transiently bound with CLIP prior to its binding with an antigenic peptide [26]. As the antigenic peptides that bind to the MHC-I molecules are typically derived from proteins of cytosolic origin, and also because only the iTCR^I^ proteins (which are present inside the lumen of RER) have the ability to selectively bind the antigenic peptides, it is thus believed that the iTCR^I^ proteins will work closely with the PLC. Specifically, it is speculated that an iTCR^I^ protein may form an initial binding complex with the antigenic peptide that is loosely bound to an MHC-I molecule. During this process, the presence of other proteins (e.g., calnexin, ERp57, calreticulin, tapasin, *β*_2_-microglobulin) [28] will jointly help complete the initial binding interaction, and more importantly, help finalize the loading of the antigenic peptide to an MHC-I molecule. An alternative possibility to the above scenario is that the antigenic peptide may bind to iTCR^I^ first to form an initial complex, and this iTCR^I^–peptide complex will then present the selected peptide to an MHC-I molecule. In either scenario, following the productive loading of the antigenic peptide, the MHC-I–peptide complex will display increased stability with high binding affinity, and thus dissociate from the PLC (including iTCR^I^). Following the dissociation, the MHC-I–peptide complex will then exit the RER, proceed to the Golgi complex and exocytic vesicles, and ultimately reach the cell surface; in the meantime, the dissociated iTCR^I^ will remain in the RER lumen to take part in the next round of antigen binding and transferring to another MHC-I molecule.

Since a pathogen may lead to the activation of many different CD4^+^ T cells, it is predicted that a somatic cell may have the ability to selectively acquire mRNAs (delivered through EVs) from different clones of activated CD4^+^ T cells. As a result, multiple versions of the iTCR^I^ proteins may be concomitantly translated and present in one somatic cell, which may beneficially facilitate the presentation of multiple antigenic peptides. In so doing, it may also help increase the efficiency of antigen presentation by pathogen-infected somatic cells.

Understandably, the activated CD4^+^ T cell-derived mRNAs which are ectopically expressed in MHC-I-expressing somatic cells will be different from the naïve CD4^+^ T cell-derived mRNAs which are expressed in APCs, as these two mRNAs will be translated into two different intracellular TCR proteins (i.e., **iTCR^I^** and **iTCR^II^**) with different subcellular localizations—despite the fact that they may share the same peptide-binding site structure. While the iTCR^I^ proteins in infected somatic cells are destined for their joint functioning with the MHC-I molecules, the iTCR^II^ proteins in APCs are functionally coupled with the MHC-II molecules in a different subcellular compartment. It is speculated that the iTCR^I^ and iTCR^II^ proteins contain specific peptide sequences in their structures which will selectively guide them to the right subcellular locations to perform their specialized functions.

Here, it is worth noting that the total numbers of different iTCR^II^s present in all APCs in the body are expected to be enormous, essentially the same as the entire TCR repertoire of all naïve CD4^+^ T cells produced in the body. In contrast, the total numbers of different iTCR^I^s present in somatic cells are usually very small, as only those TCRs of fully activated CD4^+^ T cells (which have high binding affinity for the invading pathogens’ antigenic peptides) are selected.

After the pathogen-invaded somatic cells receive EVs released from the activated CD4^+^ T cells and start to express the iTCR^I^ proteins, these somatic cells will be equipped with the ability to selectively present the antigenic peptides on their cell surfaces in complex with the MHC-I molecules, and thus are ready for selective targeting by the CD8^+^ cytotoxic T cells.

Here, it should be noted that not all somatic cells will automatically have the ability to receive EVs released by activated CD4^+^ T cells. It is speculated that only those “activated” somatic cells will upregulate the expression of MHC-I molecules and other required costimulatory proteins (likely CD80/CD86), along with the proteins specifically required for receiving the EVs produced by activated CD4^+^ T cells. It is highly possible that activation of nucleated somatic cells may be triggered by the presence of the invading pathogens inside these cells, which then starts to release relevant cytokines for their own activation as well as for the activation of the neighboring cells.

Based on the proposed mechanistic explanation, it is understood that because the iTCR^I^ proteins expressed in many somatic cells have already gone through rounds of stringent selection to avoid cross-reactivity against self-antigens, these iTCR^I^ proteins have little or no chance of randomly selecting any non-antigenic self-peptides for MHC-I presentation. However, here it should also be noted that the selective presentation of only antigenic (mostly foreign) peptides by iTCR^I^ and MHC-I molecules does not mean that self-peptides are not presented by MHC-I molecules on the cell surface of normal cells. In fact, there is experimental evidence indicating that non-antigenic self-peptides can be presented in complex-with MHC-I molecules on the surface of normal cells. First, studies have shown that normal cells can present certain cellular peptides on their cell surface through MHC-I molecules [29,30]. Second, it is known that certain types of cancer cells will try to evade the immune system by removing the MHC-I molecules on their cell surface such that they can avoid presenting cancer-specific antigens [31]. Third, natural killer cells have the ability to identify and destroy those cells (normal or cancerous) that lack MHC-I molecules on their surface [32]. Lastly, from a theoretical point of view, most somatic cells in the body are pathogen-free and non-cancerous, and if MHC-I molecules cannot present self-peptides, then it would mean that very few or no MHC-I molecules will be present on the surface of normal cells (as an empty MHC-I molecule without a peptide normally will not be presented on the cell surface). If this were the case, then many normal cells in the body likely would be constantly facing the deadly attacks by natural killer cells. In fact, we know that this is untrue, which would mean that normal cellular peptides likely are presented on the surface of normal cells in complex with MHC-I molecules, for the maintenance of normal immune system functions. To fulfill this particular function, it is speculated that there is a small group of ‘generic’ self-peptides (with very limited varieties) that are specially produced in many normal somatic cells, and these peptides have no cross-reactivity with the thymus-selected TCRs (and thus also iTCR^I^s). Here, it is further speculated that there must be a cellular protein (or a few proteins) which is functionally very similar to the iTCR^I^ proteins, and these proteins can selectively bind the generic, non-antigenic self-peptides and then deliver them to MHC-I molecules for presentation on the surface of normal cells.

Lastly, it should be noted that earlier studies have shown that the CD4^+^ T cells are highly capable of producing and releasing exosomes to regulate the body’s immune system functions [33,34,35]. Additionally, earlier studies have reported that there is an ectopic expression of TCR-like immune receptors in non-T cells in humans, such as neutrophils and macrophages [36,37,38,39,40,41]. Interestingly, the ectopic expression of TCR-like immune receptors in neutrophils in humans was found to be present across the entire life span, although the repertoire diversity declines in old age [38]. Additionally, TCR-*αβ* was detected in macrophages in atherosclerosis lesions [38] and tumor microenvironments [33]. Similarly, TCR-*αβ* bearing macrophages accumulate at the inner host–pathogen contact zone of caseous granulomas from patients with lung tuberculosis [37].

Interestingly, two recent studies [42,43] further reported that the TCR-*αβ* proteins are found in many cell types of the male and female reproductive systems of BALB/c mice, such as vaginal cells, fallopian tubular cells, cumulus cells, epithelial cells and sperms. The levels of TCR-*αβ* proteins in some of the cells appear to be regulated according to the estrus cycle [42,43]. Notably, the MHC proteins were also jointly found in some of the cells [42,43], and their levels became almost undetectable during pregnancy [42,43].

One possible explanation for the above observations is that these ectopically expressed TCR-*αβ* proteins may be associated with certain unique biological functions, i.e., they may be the intracellular TCR proteins (namely iTCR^I^ or iTCR^II^) as proposed in this paper, and their mRNAs are produced by CD4^+^ T cells. Alternatively, it may be argued that due to the extensive T-cell activation and stress under the pathogenic conditions as described in some of the above studies, T cells may be cleared by the scavenging macrophages, along with their mRNAs. While this possibility is not completely impossible, it appears less likely in light of the fact that the TCR proteins were readily detected in cells of the male and female reproductive systems as these regular somatic cells usually do not have the ability to phagocytose other cells including T cells or their debris. A more likely explanation is that the presence of TCRs in different somatic cells (including cells of the male and female reproductive systems) is a more general phenomenon, which may reflect a more specific mechanism as postulated in this paper. It is hoped that further experimental studies may help verify these potential possibilities.

**Figure 2:**
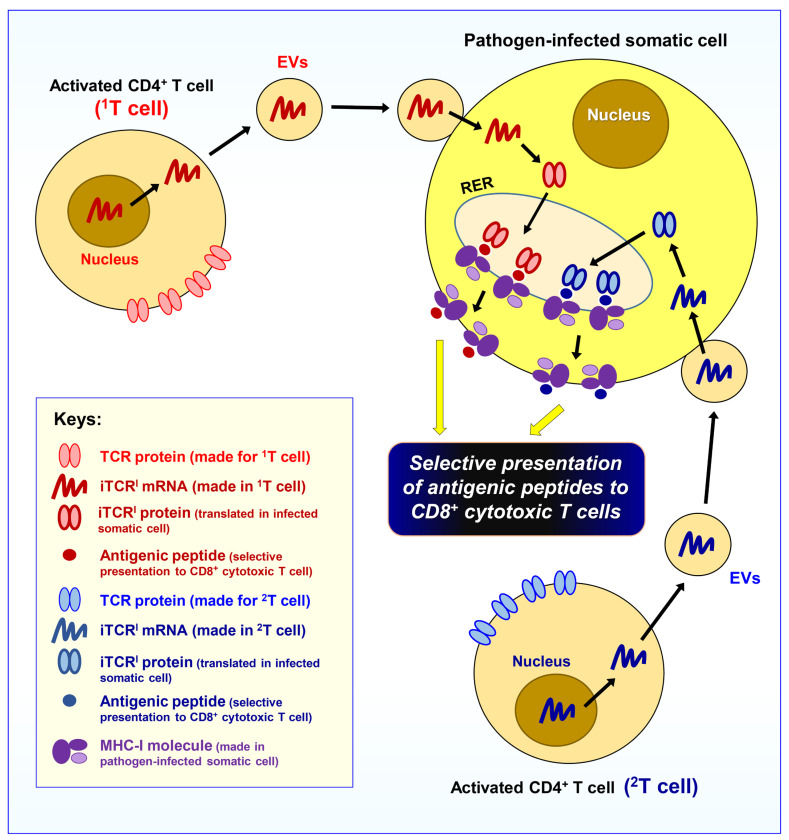
**Hypothesis II. The activated effector CD4^+^ T cells will release extracellular vesicles (EVs) specially designed to arm pathogen-invaded somatic cells for antigen presentation.** As depicted, it is hypothesized that a subset of the activated effector/helper CD4^+^ T cells will release EVs which specifically target pathogen-infected somatic cells in the body, and these EVs contain different mRNAs that will be translated into another intracellular version of the same TCR (**iTCR^I^**). The iTCR^I^ proteins will work jointly with the MHC-I molecules for the selective presentation of antigenic peptides to the CD8^+^ cytotoxic T cells. Note that an infected somatic cell usually can receive the EVs from many different clones of activated CD4^+^ T cells (in the figure, two representative activated CD4^+^ T cells, i.e., **^1^T** and **^2^T** cells, are drawn); as a result, the pathogen-infected somatic cells can present multiple different antigenic peptides (derived from the pathogens) on their surface.

In summary, as depicted in Figure 2, it is hypothesized that the fully activated T cells will release EVs which contain mRNAs and will selectively fuse with pathogen-infected somatic cells in the body for translation into iTCR^I^ proteins. In so doing, it will prepare pathogen-infected somatic cells to selectively present the invading pathogens’ antigenic peptides on their cell surface in complex with the MHC-I molecules to prepare for attacks by CD8^+^ cytotoxic T cells. Although there are many different subtypes of CD4^+^ effector/helper T cells in the body, it is presently unclear which subset(s) of the CD4^+^ effector/helper T cells actually fulfill this unique function of producing EVs to selectively arm pathogen-invaded somatic cells.

### 2.4. A Subset of Activated CD4^+^ T Cells Will Become Other Helper T Cells

The presentation of a pathogen-derived antigenic peptide by an APC, irrespective of where the antigenic proteins come from (either from a virus, bacterium, worm or parasite, or others), all utilizes the same mechanism, i.e., the antigenic peptide from a pathogen is presented to a naïve CD4^+^ T cell by an APC in complex with its MHC-II molecules. After that, the activated CD4^+^ naïve T cell will return to the thymus to ensure that it does not cross-react with any self-peptides, and then a fraction of the activated CD4^+^ T cell will leave the thymus and become clones of effector/helper T cells of different subtypes/subsets. As discussed above, some of the activated CD4^+^ T cells will then release EVs (which contain iTCR^I^ mRNAs) to arm infected somatic cells with the ability to selectively present pathogen-derived antigenic peptides. As discussed below, there are other subsets of CD4^+^ effector/helper T cells, which will work jointly and in concert in the fight against the invading pathogens.

Because different invading pathogens survive in different cellular environments (some survive inside the cells, whereas others survive outside the cells), different types of immune responses are selectively activated to deal with each type of the pathogens. Understandably, in order for the body to mount an effective immune response against a certain type of invading pathogens, different subsets of the helper T cells are selectively formed depending on the types of the invading pathogens and also on the types of immune response needed. Here, the question is: How does an activated CD4^+^ T cell know all these beforehand so that it can undergo the selective differentiation to form the right subset(s) of effector/helper T cells to precisely aid the immune response against the invading pathogens? Here, it is speculated that a naïve CD4^+^ T cell likely is effectively informed by an APC at the time of antigen presentation regarding the type of pathogens from which the antigenic peptide is derived and the exact type of APC which is presenting the antigen to the naïve CD4^+^ T cell. As we know, an APC will release “polarizing cytokines” (which constitute Signal-3), and these cytokines, along with Signal-1 and 2, will selectively alter the gene expression pattern of a CD4^+^ T cell being activated. Hence, the unique combination of Signal-1, 2 and 3 will dictate the future fate of this clone of T cells, i.e., which subset(s) of the effector/helper T cells they will develop into. During this process, the neighboring innate immune cells may also aid in informing the naïve CD4^+^ T cell about the type(s) of the invading pathogens via the unique cytokines they release.

Here, let us use B cells as an example to illustrate in more detail the proposed mechanism as to how a helper T cell can selectively activate the function of a specific B cell (or B cell clone). Assume that there is a specific B cell (or B cell clone) in the body which is labeled as **^1^B cell**. This ^1^B cell serves as a professional APC and has just completed presenting the antigen (from an invading pathogen) to a CD4^+^ naïve T cell (labeled as **^1^T cell**). Here, it should be noted that because the naïve ^1^T cell is selectively activated by the ^1^B cell, it means that the ^1^B cell contains the intracellular iTCR^II^ proteins which are also present on the surface of the ^1^T cell; otherwise, the ^1^T cell will not be able to selectively recognize the antigenic peptide presented by the ^1^B cell in complex with its MHC-II. During the antigen presentation by ^1^B cell to naïve ^1^T cell, cytokines are released from ^1^B cell for the activation of ^1^T cell. These polarizing cytokines (Signal-3), along with the binding of its TCR to an antigenic peptide (Signal-1) and the engagement of costimulatory ligands present on ^1^B and ^1^T cells (Signal-2), will lead to the selective activation of the ^1^T cell and induce a signature change in its gene expression which will determine its highly specified fate. During the process, this ^1^T cell is informed of which type of professional APCs has presented the antigenic peptide to it, and to which family the pathogen belongs. Assume that the fully activated CD4^+ 1^T cell now has successfully gone through the double checking in the thymus and is becoming a subset of effector/helper T cells, these cells can precisely recognize and thus help the ^1^B cell (or its clone) but likely not other professional APCs. Since the B cells have so many different clones, here another question arises: how does the helper ^1^T cell (or its clone) knows to selectively activate the ^1^B cell (or its clone)? The explanation is rather straightforward based on the proposed mechanism: as the intracellular iTCR^II^s in the original ^1^B cell have the same peptide-binding site as the TCRs of the helper ^1^T cell, naturally the ^1^T cell can selectively recognize the ^1^B cell through the antigenic peptides presented by the ^1^B cell (in complex with its MHC-II molecules). Here, it is of interest to note that when the CD4^+ 1^T cell encounters the ^1^B cell the first time and the TCR of the ^1^T cell identifies the peptide presented by the MHC-II molecule of the ^1^B cell, this first encounter serves the function of selective antigen presentation from the ^1^B cell to the naïve CD4^+ 1^T cell. However, when the activated ^1^T cell becomes a helper ^1^T cell, the second encounter between the helper ^1^T cell and ^1^B cell serves the function of selective activation of the ^1^B cell (or its clone). Following this activation, the ^1^B cell will undergo cell division to increase its numbers, and this ^1^B cell clone will then perform a number of immunological functions in the fight against the invading pathogens. Therefore, it is understood that within the same subgroup of helper T cells (e.g., T_H_1 cells, which have numerous T_H_1 clones), each clone of the helper T_H_1 cells (such as the ^1^T_H_1 clone) will only selectively activate a specific clone of the other immune cells (such as the ^1^B cells); these two clones of cells are selectively coupled because they share the same TCR structure and can bind the same antigenic peptide. Although the initial naïve CD4^+ 1^T cell in this case is activated by ^1^B cell, it should be noted that the activated ^1^T helper cells may also activate other APCs that contain the same iTCR^II^, besides the ^1^B cell (or its clone).

Additionally, the helper ^1^T cells may have other subsets, and they will jointly regulate the complex functions of body’s immune system. For instance, different B cells can be activated to produce different antibodies (e.g., IgG and Ig E), and they have different functions in defending pathogens of different groups (some live inside the host cells, and some live outside the host cells). It is likely that different helper T cell subsets are involved in regulating different B cells to produce different Ig isotypes. For instance, the T_H_1 cells can promote B cells to produce antibodies that contribute to cell-mediated immunity (e.g., isotypes like IgG2a that can “arm” NK cells for cytotoxicity), whereas T_H_2 cells promote B cells to produce antibodies that mediate the clearance of extracellular pathogens (e.g., isotypes like IgE that induce the release of molecules that harm extracellular parasites).

### 2.5. A Subset of Fully Activated CD4^+^ T Cells Will Be Transformed into Naïve CD8^+^ T Cells

As discussed above, after a naïve CD4^+^ T cell is fully activated by an APC, it will travel back to the thymus for another round of negative selection. It is hypothesized that while still in the thymus (after passing the selection), a fraction of the fully activated CD4^+^ T cells will be transformed into naïve CD8^+^ T cells. During this transformation, it is expected that critical changes in genomic organization and gene expression will take place in the CD4^+^ T cells, resulting in the silencing of the *CD4* gene plus the expression of genes associated with the CD8^+^ T cell lineage. For a CD4^+^ T cell, Th-POK activates *CD4* expression and inhibits the expression of genes that control CD8^+^ T cell differentiation, including genes encoding CD8 itself and Runx3 [44]. Reciprocally, Runx3 activates *CD8* expression in CD8^+^ T cells, while inhibits the expression of genes encoding CD4 and Th-POK.

While the newly formed naïve CD8^+^ T cells are still in the thymus, they will undergo the negative selection for their potential reactivity against any self-peptides presented by mTECs in complex with the MHC-I molecules. Note that this is actually the first time for the single-positive CD8^+^ T cells to undergo the negative selection against MHC-I-presented self-peptides. Understandably, this negative selection for the CD8^+^ T cells will have a very high success rate compared to the CD4^+^ T cells (which happened prior to their first egress from the thymus) as the TCRs in a naïve CD8^+^ T cell are the same as the TCRs in a fully activated CD4^+^ T cell which has already gone through the negative selection process twice against self-peptides presented by thymic MHC-II molecules.

According to the proposed hypothesis, it is speculated that all naïve CD8^+^ T cells in the body are derived from activated CD4^+^ T cells; these cells have already had their TCRs selected for their ability to identify antigenic (mostly foreign) peptides but not non-antigenic self-peptides. From a theoretical point of view, this mechanism confers a great advantage of having both the CD4^+^ effector/helper T cell clone and the CD8^+^ cytotoxic T cell clone share the same TCRs on their surface, thus ensuring that both groups of T cells can recognize the same type of the antigenic peptides with the same selectivity and affinity. Furthermore, since pathogen-infected somatic cells are armed by activated CD4^+^ T cells to express the intracellular iTCR^I^ proteins (which share the same antigen-binding site structure as the cell surface TCRs of both CD4^+^ and CD8^+^ T cells), this will further ensure that the antigenic peptides presented by pathogen-infected somatic cells can be precisely recognized and targeted by the TCRs of the CD8^+^ T cells.

Here, it is of interest to note that mTECs have an extraordinary ability to express proteins from all over the body, most notably, the tissue-specific antigens [45,46,47]. These proteins will be chopped into pieces in the right subcellular compartment(s) for presentation by both MHC-I and MHC-II molecules at the mTECs’ surface for T cell selection [48,49]. Additional studies found that mTECs express a unique protein, AIRE, which allows these cells to express proteins that are ordinarily found only in other organs or cells [50,51]. Mechanistically, AIRE does not bind to DNA directly; rather, it binds to epigenetic marks on histones associated with closed chromatin. AIRE then recruits transcription factors to these silenced gene promoters, allowing RNA polymerase to gain access. Recent studies indicate that AIRE is not the only transcriptional regulator in mTECs that controls expression of all the proteins (including those tissue-specific antigens). In addition, other proteins, such as members of the FEZF2 zinc finger proteins, may play a similar role [52,53]. Hence, mTECs have a very unique role in assisting the screening of the developing T-cell repertoire and ridding it of self-reactive T cells.

There is some indirect experimental evidence that offers partial support for the hypothesis that all naïve CD8^+^ (single positive) T cells may come from activated CD4^+^ T cells. Based on the proposed hypothesis, it is understood that the TCR repertoire of the CD8^+^ T cells in the body will be very limited compared to the total repertoire of all naïve CD4^+^ T cells, as the repertoire of all naïve CD4^+^ T cells will include all the possibilities whereas the CD8^+^ T cell repertoire will only include those fully activated CD4^+^ T cells. This prediction is supported by several earlier studies [54,55,56,57,58,59]. For instance, when the TCR-*β* repertoire in CD4^+^ and CD8^+^ T cells from 30 healthy donors were analyzed for their clonal patterns, it was found that the CD4^+^ TCR-*β* repertoire has far more counts and diversity than CD8^+^ TCR-*β* [56,57]. In addition, the CD8^+^ TCR-*β* repertoire of a person is actually a lot more similar to other people’s CD8^+^ TCR-*β* repertoires, but it is not similar to this person’s own CD4^+^ TCR-*β* repertoire. Despite this unique and unexpected finding, cluster pattern analysis still clearly shows that the CD4^+^ and CD8^+^ TCR-*β* repertoires of the same person have a far higher degree of connectedness as compared to the CD4^+^ and CD8^+^ TCR repertoires of different individuals. Additionally, previous analyses have shown that although people progressively lose naïve CD8^+^ T cells with age, the absolute numbers of naïve CD4^+^ T cells in peripheral blood remain stable [60,61]. All these unique features concerning the populations of CD4^+^ and CD8^+^ T cells in the body are actually in line with the hypothesis that all naïve CD8^+^ T cells are originally derived from fully activated CD4^+^ T cells.

It is known that during the initial thymocyte maturation inside the thymus, the thymocytes express both CD4 and CD8 proteins, becoming the CD4^+^CD8^+^ double-positive T cells. The purpose of having the early thymocytes to express both CD4 and CD8 proteins is simply for testing whether both proteins can effectively identify and bind MHC-II and MHC-I molecules, respectively, with an acceptable binding affinity (i.e., the process of “positive selection”). Only those thymocytes that are capable of recognizing the self MHC-II and MHC-I molecules are positively selected. Most (>90%) of the CD4^+^CD8^+^ double positive thymocytes will fail this positive selection process and then undergo cell death. After this step, it is believed that the CD4^+^CD8^+^ double positive thymocytes will lose their CD8 expression and become the CD4^+^ (single positive) thymocytes, which, after successfully passing the negative selection, will emigrate out of the thymus as the naïve CD4^+^ T cells. In this paper, it is hypothesized that all CD8^+^ (single positive) naïve T cells present in the thymus are actually derived from the returning fully activated CD4^+^ T cells. While still in the thymus, the newly formed naïve CD8^+^ T cells will permanently lose their CD4 expression but will start to express CD8 as well as other related lineage markers, and they will also be negatively selected one more time (the third time) to minimize the possibility that they may incidentally bind to any self-peptides presented by the mTECs in complex with the MHC-I molecules.

After a newly formed naïve CD8^+^ T cell has successfully passed the negative selection in the thymus, it will exit the thymus. After leaving the thymus and moving to the secondary lymphoid tissues in the periphery, a naïve CD8^+^ T cell is still functionally immature and does not have the ability to kill pathogen-infected target cells; this CD8^+^ T cell is commonly referred to as a cytotoxic T lymphocyte precursor (CTL-P). Only after a CTL-P is fully activated by a licensed DC, then it will undergo differentiation and growth to form a clone of mature CTLs with cytotoxic functions. The mechanism underlying CTL-P activation by a licensed DC is briefly described below.

*Mechanism of activation of a naïve CD8^+^ T cell.* The activation of a naïve CD8^+^ T cell (CTL-P) involves multiple steps and requires multiple activation signals. The first critical step involves DC licensing. In the absence of DC licensing, a newly formed naïve CD8^+^ T cell can not be activated. In the literature, it is generally held that the process of DC licensing involves the enabling of a DC to jointly express both MHC-I and MHC-II molecules, and after that, the licensed DC will acquire the ability to “cross-present” antigens through both MHC-I and MHC-II molecules [62,63,64].

It has been suggested that a DC can be licensed in several ways—either by a CD4^+^ helper T cell (usually of the T_H_1 subset) or by engagement with microbial products, which activate the Toll-like receptors. However, based on the hypothesis proposed in this paper, it is understood that even if a DC expresses both MHC-I and MHC-II molecules, and even if a DC itself is infected with the pathogens, this DC still cannot selectively present the antigenic peptides (derived from the pathogens)—unless it express both iTCR^I^ and iTCR^II^ proteins such that it can selectively present the antigenic peptides by its MHC-I and MHC-II molecules. Accordingly, it is hypothesized that for DC licensing, a CD4^+^ helper T cell needs to supply iTCR^I^ mRNAs for the DC. Here the question is: how does the CD4^+^ helper T cell selectively deliver the iTCR^I^ mRNAs to the intended DC? If we assume that a particular DC has already received the cognate iTCR^II^ mRNAs earlier through a naïve CD4^+^ T cell, now the cognate CD4^+^ helper T cell (which is derived from the original naïve CD4^+^ T cell) can still recognize the antigenic peptide being presented by this DC (in complex with the MHC-II molecules). This interaction between a CD4^+^ helper T cell and a DC likely will form intimate contacts between these two cells, usually through the formation of an immunosynapse. As a result of this close contact, the iTCR^I^ mRNAs will be selectively delivered from a helper T cell to a DC, and then the DC will start to express both iTCR^I^ and iTCR^II^ proteins of the same type. An alternative possibility to the above scenario is that a newly formed naïve DC may be used for the particular purpose of “licensing” by a CD4^+^ helper T cell. During this process, it is believed that the T cell still needs to form an immunosynapse with the DC such that the mRNAs for the cognate iTCR^I^ and iTCR^II^ (from the same helper T cell) will be selectively delivered to the DC. Moreover, after this naïve DC receives the cognate iTCR^I^ and iTCR^II^ mRNAs, changes will take place in the DC which will disallow receiving similar mRNAs from other clones of the CD4^+^ helper T cells.

Here, it is important to point out that for DC licensing, a DC should not concomitantly express multiple copies of the iTCR^I^ or iTCR^II^ proteins from different clones of the helper T cells. The reason for this requirement is rather simple: if different types of iTCR^I^ or iTCR^II^ proteins are jointly present in a licensed DC, it will lead to the presentation of multiple antigenic peptides on the surface of a single DC, which will then further lead to the accidental cross-activation of other CD8^+^ T cells and thus the potential development of autoimmunity.

Based on the above explanations, it is understood that one of the most crucial functions of the CD4^+^ helper T cell during DC licensing is to deliver the iTCR^I^ mRNAs for the DC such that the licensed DC will have the ability to jointly express cognate iTCR^I^ and iTCR^II^ proteins simultaneously. Additionally, the CD4^+^ helper T cell will produce cytokines (e.g., IFN-γ) that can activate the DC, and will also express CD40 ligand (CD40L, also known as CD154), which provides a crucial costimulatory signal to the DC. CD40L is recognized by CD40, a TNF receptor family member expressed by an activated DC. When CD40L binds to CD40, CD40 will initiate a signaling cascade in the DC which increases the expression of costimulatory ligands (CD80 and CD86), chemokines and cytokines; all these factors are crucially required during the process of activating the CD8^+^ CTL-Ps.

After a DC is licensed (assuming that it is still tightly connected to a CD4^+^ T cell through an immunosynapse), it can cross-present the same cognate peptides through its MHC-I and MHC-II molecules. Since the MHC-I-presented peptides on the surface of a DC can be readily recognized by the cognate TCRs of a CD8^+^ CTL-P, this will lead to the binding interactions between the CTL-P and DC, thus resulting in the formation of a three-cell complex, containing a DC, a CD4^+^ helper T cell, and a CD8^+^ CTL-P. The formation of this three-cell complex is a characteristic step during the activation of a CD8^+^ CTL-P. The major activation signals for a CD8^+^ CTL-P usually include: ***i***. Signal-1 is the antigen-specific signal transmitted by the TCR complex upon recognition of an MHC-I–peptide complex on an “licensed” DC. ***ii***. Signal-2 is the costimulatory signal which is transmitted when CD28 on the CTL-P surface binds CD80/86 (B7) on the DC. ***iii***. Signal-3 is the signal provided by the CTL-P’s high-affinity binding to IL-2 receptor by IL-2, which is generated by the helper T cell and also the engaged CTL-P itself. These three signals induce the proliferation and differentiation of the antigen-activated CTL-Ps into effector CTLs and memory cells.

The fully activated CD8^+^ CTLs will then turn on the expression of a number of proteins, including granzyme B and perforin, which are packaged into lytic granules. These CD8^+^ CTLs can induce the death of pathogen-infected somatic cells via two mechanisms: the perforin-granzyme pathway and the Fas–FasL pathway [65,66]. Rather than inducing cell lysis, both processes induce the target cell to undergo apoptosis, typically within an hour of contact with the CD8^+^ CTLs. CTL killing involves a carefully orchestrated sequence of events that begins when the attacking cell binds to the target cell and forms a cell–cell conjugate. Formation of a CTL–target cell conjugate is followed within several minutes by a Ca^2+^-dependent, energy-requiring step in which the CTL induces death of the target cell. The CTL then dissociates from the target cell and may go on to bind another target cell.

Lastly, it is of interest to note that the population of the effector CD8^+^ T cells is usually far more abundant than the population of the effector CD4^+^ T cells. This phenomenon is readily understood in the light of the proposed hypotheses. Given that during an infection (either with viruses or bacteria), a lot of host cells are usually infected. As a result, a very large number of mature and functional CD8^+^ CTLs are needed to clear up the infected cells. In comparison, the number of required CD4^+^ helper T cells likely will be much smaller compared to the CD8^+^ CTLs as the CD4^+^ T cells will produce and release EVs to help arm the pathogen-infected somatic cells. Since one CD4^+^ helper T cell can release a large number of EVs that may arm many infected somatic cells, so the total number of CD4^+^ T cells required for this function is expected to be much smaller than the total number of CD8^+^ T cells.

In summary, the conversion of a naïve CD8^+^ T cell (CTL-P) to a CTL requires engagement with a licensed DC for activation. For DC licensing, it is hypothesized that the DC needs to selectively receive the iTCR^I^ and iTCR^II^ mRNAs from the same clone of T cells. As such, the licensed DC can selectively express two intracellular versions of the same TCRs (i.e., iTCR^I^ and iTCR^II^), which will enable the DC to cross-present the same type of antigenic peptides through its MHC-I and MHC-II molecules. As a result, the licensed DC will be able to activate the CD8^+^ CTL-P through its joint and concomitant interactions with a CD4^+^ helper T cell and a CD8^+^ CTL-P.

### 2.6. Mechanism for the Formation of CD4^+^ Regulatory T Cells

The CD4^+^ regulatory T cells (T_REG_) was first described in the late 1960s [67]. The T_REG_ cells play a crucial role in immunosuppression, ensuring tolerance to self-antigens, innocuous allergens and commensal microflora [68,69,70,71,72]. These T_REG_ cells express FoxP3, a lineage-defining transcription factor [11,73,74,75], along with some other signature genes, such as CTLA-4 and high levels of the IL-2R α chain (CD25) [75,76,77,78,79,80].

How are the CD4^+^ T_REG_ cells formed in the body? In the literature, it is generally thought that a fraction of the thymocytes that experience high-affinity TCR interactions in the thymus do not die by negative selection but instead develop into T_REG_ cells. A number of mechanisms have been suggested in the past trying to explain why these T cells, whose TCRs display high affinity for self-antigens in the thymus, are not eliminated by negative selection [81]. The best-known model is the “hit and run” hypothesis, which suggests that high-affinity but transient engagement of a T cell’s TCR with the MHC–antigen complex in the thymic medulla favors the generation of T_REG_ cells; however, more sustained, high-affinity TCR engagement in the thymus favors deletion of the self-reactive lymphocytes (via elimination).

In light of the newly proposed hypotheses, a different explanation is entertained here. Specifically, it is speculated that a few different scenarios may exist, which will all lead to the generation of the CD4^+^ T_REG_ cells:

***Scenario 1.*** *A fully activated but self-reactive CD4^+^ T cell will be converted into a clone of T_REG_ cells.* After a naïve CD4^+^ T cell is fully activated by an APC and then returns to the thymus to undergo another round of negative selection, and if it happens that the TCRs of the activated CD4^+^ T cell can cross-react with a self-antigen presented by the thymic cells (mostly mTECs) in complex with the MHC-II molecules, then this CD4^+^ T cell will be converted into a T_REG_ cell, which will then undergo proliferation (in the thymus initially and later in secondary lymphoid tissues) to form a T_REG_ clone specific for this self-antigen. It is believed that FoxP3, along with other signature genes, will be activated in this clone of self-reactive CD4^+^ T cells while they are still in the thymus.

Here, there are two potential possibilities with the TCRs of this fully activated CD4^+^ T cell: one is that its TCRs indeed can recognize the invading pathogen’s peptide, but they can also cross-react with a self-antigen. The other possibility is that its TCR actually cannot recognize the pathogen’s peptide but just happens to cross-react with a self-antigen. In either case, it will be detrimental to the host if this CD4^+^ T cell is allowed to develop into mature CD4^+^ effector T cells and cytotoxic CD8^+^ T cells. Instead, this fully activated CD4^+^ T cell will be converted into a clone of T_REG_ cells (and memory T_REG_ cells), and understandably, their function is to suppress autoimmunity against this particular self-antigen in the peripheral tissues. This subset of CD4^+^ T_REG_ cells may be somewhat similar in function to the tissue-resident thymic T_REG_ cells (tT_REG_ cells).

In the literature, there is another subset of CD4^+^ T_REG_ cells, called peripheral T_REG_ cells (pT_REG_ cells). It has been suggested that the pT_REG_ cells may arise from CD4^+^ T cells that are activated in secondary lymphoid tissues after antigen exposure in the context of TGF-*β* and IL-10 cytokines [82]. However, in the light of the proposed hypothesis, it is less likely that the pT_REG_ cells can arise entirely in the periphery; it is more likely that these pT_REG_ cells also need to return to the thymus during their development to confirm their cross-reactivity against self-antigens presented by mTECs and then are commissioned to be pT_REG_ cells by turning on their FoxP3 gene expression along with other signature genes. In other words, there might be no real pT_REG_ cells that are generated entirely in the periphery without the need to return to the thymus prior to being commissioned.

From a mechanistic point of view, CD4^+^ T_REG_ cells may achieve immunosuppression against a specific self-antigen via a number of mechanisms; some are briefly discussed below:

First, CD4^+^ T_REG_ cells can modulate the functions of other immune cells. As mentioned earlier, Signal-2 is produced by the costimulatory ligands expressed on APCs which then interact with the costimulatory receptors (CD28 or ICOS) expressed on the T cells [83]. However, co-inhibitory receptors, such as CTLA-4, PD-1 and BTLA expressed on T_REG_ cells, can bind to their respective ligands on APCs [84,85]. Since these APCs express the iTCR^II^ proteins which are cognate with the TCRs on the CD4^+^ T_REG_ cells and can selectively present the same self-reactive antigen, the interaction of a CD4^+^ T_REG_ cell with an APC will result in its selective inactivation. It is hypothesized that the inactivated APC will be recommissioned and then go on to help inactivate all other naïve T cells which can recognize this particular self-reactive antigen presented by the APCs. Stated differently, those APCs that are inactivated by a T_REG_ cell are commissioned to selectively inactivate all naïve CD4^+^ T cells and CD8^+^ CTL-Ps whose TCRs can recognize this self-antigen. The inactivated CD8^+^ CTL-Ps will become anergic [86]. Offering partial support for this suggestion, earlier studies have shown that the T_REG_ cells can effectively reduce the expression of various costimulatory molecules on APCs, but not MHC-I or MHC-II [83]. Notably, it is also known that T_REG_-mediated immunosuppression is highly antigen specific. This is readily understood in the light of the proposed hypothesis as the T_REG_ cells will only inactivate those APCs that can selectively express the cognate iTCR^II^ proteins and thus can present the same self-reactive antigen. In addition, the CD4^+^ T_REG_ cells have also been shown to directly kill other immune cells (such as CD8^+^ effector T cells), by means of granzyme and perforin [87].

Second, many studies have shown that CD4^+^ T_REG_ cells can suppress the immune system functions in a rather nonspecific manner [88]. For instance, T_REG_ cells can secrete inhibitory cytokines (e.g., IL-10, TGF-*β* and IL-35), suppressing the activity of other nearby T cells and APCs. Additionally, as T_REG_ cells express high levels of CD25 (the high-affinity IL-2 receptor), they can act as a sponge, absorbing this growth- and survival-promoting cytokine and further discouraging expansion of local immune-stimulatory effector T cells [85].

***Scenario 2.*** *A partially activated but self-reactive CD4^+^ T cell will also become a clone of T_REG_ cells.* If a naïve CD4^+^ T cell’s TCR is engaged and partially activated by an APC, i.e., in the presence of Signal-1 but in the absence of Signal-2 (i.e., the costimulatory signal, such as CD80/CD86), this situation will indicate to the T cell that the antigenic peptide presented by an APC in complex with its MHC-II may not come from an invading pathogen. During PRR-mediated engulfment of the invading pathogens by a professional APC, PRR activation will lead to enhanced antigen-presenting activity through up-regulation of MHC-II molecules and costimulatory ligands. As such, when the costimulatory ligands are absent in the APCs, it is quite possible that the peptide being presented by an APC may not come from the invading pathogens.

It is speculated that when a CD4^+^ naïve T cell is partially activated in the absence of costimulatory ligands, this CD4^+^ naïve T cell will also need to return to the thymus to go through another round of negative selection to determine whether this T cell can react with any self-peptides presented by mTECs. If it does, then it would mean that this CD4^+^ naïve T cell indeed expresses self-reactive TCRs—this T cell might have escaped the negative selection and left the thymus during its initial maturation/selection process. Should this be the case, then it is understood that this self-reactive CD4^+^ T cell also needs to be activated to become a clone of CD4^+^ T_REG_ cells; they will serve the function of suppressing future activation of other auto-reactive naïve CD4^+^ T cells and CD8^+^ CTL-Ps that express the same TCRs and can recognize the same self-peptide. The mechanisms by which these T_REG_ cells suppress the immune system functions are expected to be essentially the same as summarized under *Scenario 1*.

***Scenario 3.** A partially activated CD4^+^ T cell with no self-reactivity may become a clone of helper T cells that will mediate autoimmunity or anticancer immunity.* For a partially activated CD4^+^ T cell which does not cross-react with any of the self-peptides presented by mTECs, there are a few possibilities: ***i.*** This peptide may come from a transformed or cancer cell. ***ii.*** This peptide may come from a regular somatic cell, but its cellular components (such as proteins) are covalently modified by other chemicals and thus become antigenic. Or, this antigen may come from a somatic cell which produces a mutant antigenic protein, resulting from somatic DNA mutation.

If the antigenic peptide comes from a transformed or cancer cell, this partially activated CD4^+^ T cell after leaving the thymus likely will further encounter the same MHC-II-complexed antigen in the periphery, and thus its activation by this antigen will be further confirmed. It is speculated that following the further activation, this CD4^+^ T cell may need to move back to the thymus again to confirm that it does not cross-react, even with low affinity, with any of the mTCE-presented self-antigens. After that, this clone of activated T cells will become a special subset of helper T cells, which will play an important role in mediating the anticancer immunity. Here, I will not further speculate on the subsequent processes involved, but just like to suggest that the anticancer immune response may take much longer time to confirm and execute compared to the anti-pathogen immune response as the cancer cells in the body, unlike the invading pathogens (such as bacteria and viruses), usually develop very slowly. The body’s immune system may take much longer time and a lot more steps to make certain that the antigenic peptide is indeed from the transformed cancer cells but not from the body’s normal cells. Understandably, during the development of anticancer immune response, urgency (i.e., timeliness) would take a back seat in comparison with the anti-pathogen immune response; accuracy will be of paramount importance in the former because if a mistake is accidentally made during the hasty process, it will have very severe, even life-threatening, consequences due to the development of autoimmunity.

In a similar manner, if the antigen comes from regular somatic cells, then this CD4^+^ T cell after leaving the thymus may encounter the target peptides (in complex with MHC-II molecules) in the periphery again, and its activation by this antigen will be further confirmed. It is speculated that after this confirmation, this CD4^+^ T cell may need to go back to the thymus again to make certain that it does not cross-react, even with very low affinity, with any of the mTCE-presented self-antigens. After this step, then this activated T cells will become a special subset of helper T cells, which will, unfortunately, be involved in mediating autoimmunity against those somatic cells that produce the antigen.

Here, it is worth noting that the mechanism underlying the chronic immune rejection against an organ transplant likely is very similar to the mechanism of anticancer immunity and autoimmunity as described above. The apparent slowness in these processes is mostly because the body needs to take the necessary time to carefully perform all the checks to ensure that the antigens are indeed not the self-antigens derived from normal cellular components.

Many subsets of the CD4^+^ helper T cells exist in the body. While most CD4^+^ helper T cells are involved in fighting the invading pathogens, it is speculated that there might be a special subset of CD4^+^ helper T cells (as described above) which may mediate autoimmunity, anticancer immunity and transplant immunity. In the literature, the CD4^+^ T_H_17 cells (characterized by IL-17 production) were reported to be actively involved in autoimmunity, anticancer immunity and transplant immunity [89,90,91,92,93]. It will be of interest to determine in the future whether T_H_17 cells indeed are derived from partially activated CD4^+^ T cells as speculated above and are specialized in mediating the proposed immunological functions.

Given the fact that some of the cancer-specific antigens are often not entirely new proteins, there might be cases that some of the helper T cells later are found to have certain degrees of self-reactivity against mTEC-presented self-antigens. Should this be the case, it is then predicted that some of these CD4^+^ helper T cells may eventually be converted to T_REG_ cells, starting to express FoxP3. In fact, there were some experimental observations offering partial support for this speculation. For instance, while T_H_17 cells usually express IL-17, there are also a subset of T_H_17 cells that express both IL-17 and FoxP3, and these cells, as expected, mostly will serve as T_REG_ cells, involved in suppressing the anticancer immune responses [94,95].

In summary, when the TCRs of a fully or partially activated CD4^+^ T cell can cross-react with self-peptide(s) while it is back in the thymus, this T cell will be transformed into a clone of CD4^+^ T_REG_ cells, which will serve the function of selectively suppressing the autoimmune reactions against this class of self-antigens. Interestingly, for a partially activated CD4^+^ T cell with no self-reactivity, it is possible that this antigen may come from a transformed or cancer cell, or from a covalently modified component of a normal cell, or from a somatic cell which produces a mutant antigenic protein. It is speculated that if this CD4^+^ T cell later encounters the antigens again in the periphery, it will be further activated and transformed into a special subset of CD4^+^ helper T cells. These helper T cells will play an important role in mediating the body’s anticancer immunity or autoimmunity. However, if this clone of CD4^+^ T cells is not further activated by peripheral antigens, then they will become anergic and undergo cell death after some time.

### 2.7. Mechanism for the Formation of CD8^+^ Regulatory T Cells

Similar to the formation of CD4^+^ T_REG_ cells, it is speculated that the CD8^+^ T_REG_ cells are also formed in the thymus. Recall that it was discussed in Section 2.5 that the CD8^+^ naïve T cells are converted from fully activated CD4^+^ T cells, and afterwards, these CD8^+^ naïve T cells will undergo another round of selection in the thymus to ensure that these cells do not have any self-reactivity. However, if a CD8^+^ T cell happens to have cross-reactivity against self-peptides presented by mTECs (in complex with the MHC-I molecules), then this self-reactive CD8^+^ T cell will be developed into a clone of CD8^+^ T_REG_ cells.

From a theoretical point of view, as a CD8^+^ T cell is derived from a CD4^+^ T cell which has passed the negative selection twice in the thymus, the chances for a CD8^+^ T cell to cross-react with a self-peptide is usually far lower than the chances for a CD4^+^ T cell to cross-react with a self-peptide. Therefore, it is predicted that the total clones of CD8^+^ T_REG_ cells will be very small in the body, compared to the total clones of CD4^+^ T_REG_ cells generated in the thymus. Here, it should be noted that if a CD4^+^ T cell is found to be self-reactive in the thymus, then it will not be permitted to become a naïve CD8^+^ T cell. Similarly, a partially activated CD4^+^ T cell usually will also not be permitted to become a naïve CD8^+^ T cell.

During the normal immune response, the CD8^+^ T cells are specialized in executing the killing of pathogen-infected cells. However, when a CD8^+^ T_REG_ clone is formed, these T cells may need to single-handedly perform very diverse regulatory functions in order to effectively quell the aberrant autoimmune responses. To put it figuratively, a CD8^+^ T_REG_ cell (or clone) is somewhat like a hypothetical scenario of a lone soldier in a battlefield where he suddenly realizes that both sides of the battlefield are engaged in a wrong battle of self-destruction, whereas all other soldiers do not know. As a result, this soldier needs to stop his original role as a simple frontline foot soldier and will take on the difficult role as a multi-functional commander to stop the ongoing or pending disaster. Below are speculations on a few functions that a CD8^+^ T_REG_ cell may perform:

***i.*** It is speculated that these CD8^+^ T_REG_ cells will be specialized in suppressing the functions of licensed DC and preventing them from activating other CD8^+^ CTL-Ps, which express the same self-reactive TCRs. As discussed in Section 2.5, under usual conditions, a CD4^+^ T_H_1 cell, a licensed DC and a CD8^+^ CTL precursor will form a three-cell complex, which will lead to the activation of the CD8^+^ CTL precursor. However, when the CD8^+^ T_REG_ cell is present in the three-cell complex in place of the CD8^+^ CTL precursor, this complex will lead to joint inactivation of both CD4^+^ T_H_1 cell and licensed DC. Offering partial support for the above speculation, earlier studies have reported that CD8^+^ T_REG_ cells can inhibit the function of helper T cells, and this is a mechanism for the development of self tolerance [80,96,97]. Similarly, a CD8^+^ T_REG_ cell is known to effectively inhibit the function of the licensed DC through selective expression of certain inhibitory receptors. It is speculated that those CD4^+^ helper T cells and licensed DCs which are inhibited by the CD8^+^ T_REG_ cells will be recommissioned to acquire the ability to suppress other CD8^+^ CTL-Ps, which express the same iTCR^I^ proteins during their future encounters.

***ii.*** As the CD8^+^ T_REG_ cells have cytotoxic functions [97,98], it is speculated that these CD8^+^ T_REG_ cells may acquire the ability to selectively recognize those activated CD8^+^ CTLs and eliminate them. However, the precise mechanism by which a CD8^+^ T_REG_ selectively identifies other activated CTLs which can recognize the same antigenic peptide is presently unclear.

In summary, when a CD8^+^ T cell is found to be self-reactive while still in the thymus, it will be converted into a clone of CD8^+^ T_REG_ cells. It is speculated that these CD8^+^ T_REG_ cells will suppress the functions of both CD4^+^ T_H_1 cells and licensed DCs. These inactivated CD4^+^ T_H_1 cells and DCs will not have the ability to activate other CD8^+^ CTL-Ps that express the same self-reactive TCRs; instead, they will be recommissioned by the CD8^+^ T_REG_ cells to inactivate future CD8^+^ CTL-Ps that bear the same self-reactive TCRs. 

## 3. Some Suggestions on the Experimental Testing of the Proposed Hypotheses

To test the key hypotheses developed in this paper, many experiments may be considered. Listed below are a few initial experiments for the testing of the hypothesis depicted in Figure 1:

- Determine whether a naïve CD4^+^ T cell can release EVs which contain mRNAs encoding an intracellular version of the TCR proteins (i.e., iTCR^II^).
- Determine whether many professional APCs in the body contain mRNAs for iTCR^II^ proteins, and that these mRNAs can be ectopically expressed in APCs. It is also important to identify the protein sequences and structures of the iTCR^II^ proteins, and compare them with the original TCR protein present on the surface of T cells.
- Determine whether the iTCR^II^ proteins ectopically expressed in APCs have a subcellular localization in the late endosomes, but not on their cell surface. Furthermore, it is important to determine the mechanism by which the iTCR^II^ proteins mediate the process of selective antigen presentation by the MHC-II molecules.
- If a TCR is known to bind a peptide (complexed with an MHC-II molecule) with high affinity, we can selectively express the intracellular version of this TCR (i.e., iTCR^II^) in APCs, and then analyze what peptides are presented by the APCs. It is expected that these iTCR^II^-expressing APCs will selectively present peptides that can bind to the original TCR, but not other peptides.

Similarly, the following experiments may be considered for the initial testing of the hypothesis depicted in Figure 2:

- Determine whether the activated CD4^+^ T cells indeed can release EVs which contain different mRNAs for another version of the TCR proteins (i.e., iTCR^I^).
- Determine whether there is a widespread increase in the ectopic expression of iTCR^I^ proteins in pathogen-infected somatic cells in the body. It is also important to identify the protein sequences and structures of the iTCR^I^ proteins, and compare them with the original T-cell version of the TCR proteins.
- Determine whether the iTCR^I^ proteins ectopically expressed in pathogen-infected somatic cells have a subcellular location in the RER, but not on the cell surface. Also, it is important to determine the mechanism by which the iTCR^I^ proteins take part in mediating the selective antigen presentation by MHC-I molecules.
- We may selectively express the same type of the iTCR^I^ proteins in somatic cells. Assume that the original T-cell version of the TCR has a very high affinity for a given peptide, then we can analyze the peptides that are selectively presented by the MHC-I molecules in these iTCR^I^-expressing somatic cells. It is expected that these somatic cells will selectively present the peptides that have high affinity for the original T-cell version of the TCRs.

## 4. Concluding Remarks

**Figure 3:**
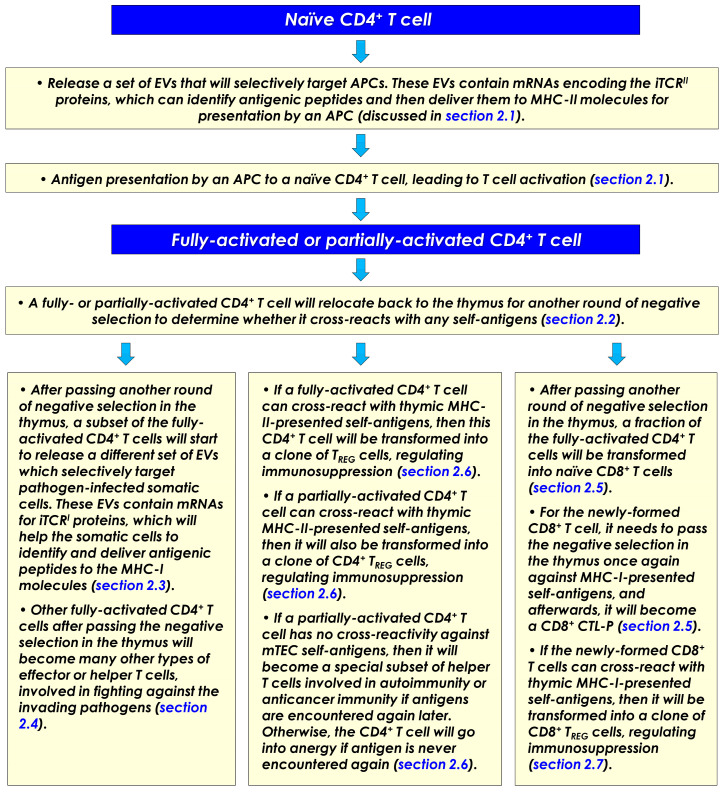
A summary of the proposed hypotheses concerning the molecular mechanisms underlying selective antigen presentation by the MHC-I and MHC-II molecules.

It is known that the MHC-I and MHC-II molecules do not have the built-in ability to distinguish self-peptides from foreign antigenic peptides, and the precise mechanism for their ability to selectively present antigenic (mostly foreign) peptides is not understood at present. As summarized in Figure 3, it is hypothesized in this paper that all naïve CD4^+^ T cells will release EVs containing mRNAs for the selective uptake by APCs. These mRNAs will be translated into the intracellular iTCR^II^ proteins which help select non-self-peptides for presentation by the MHC-II molecules. It is speculated that a naïve APC may receive EVs (which contain iTCR^II^ mRNAs) from many different naïve CD4^+^ T cells, as a way to enhance efficiency. However, once an APC starts to present an antigen, it will cease to receive additional EVs from other naïve CD4^+^ T cells. In this way, an APC normally will only present one single type of the antigenic peptides and thus will only activate one naïve CD4^+^ T cell (or its clone).

Following the full activation of a naïve CD4^+^ T cell by an APC, it is hypothesized that this T cell will return to the thymus for another round of negative selection to make certain that it does not cross-react with mTEC-presented self-peptides. After the T cell has successfully passed the negative selection, it is licensed to undergo proliferation inside the thymus and in peripheral secondary lymph tissues to become a clone of CD4^+^ effector/helper T cells. It is hypothesized that a subset of the CD4^+^ effector/helper T cells will release EVs which contain mRNAs encoding the iTCR^I^ proteins, and these EVs are specially designed for uptake by pathogen-infected somatic cells in the body. These iTCR^I^ proteins will work jointly with the MHC-I molecules to selectively present pathogen-derived antigenic peptides in infected somatic cells.

Furthermore, after the fully activated CD4^+^ T cells have passed another round of negative selection, it is hypothesized that some of the CD4^+^ T cells will be converted into naïve CD8^+^ T cells. It is speculated that all clones of naïve CD8^+^ T cells present in the body are formed this way, i.e., they all come from activated CD4^+^ T cells.

When the TCRs of a fully or partially activated CD4^+^ T cell can cross-react with self-antigen(s) while it is back in the thymus, this T cell will be transformed into a clone of CD4^+^ T_REG_ cells, which will serve the function of selectively suppressing the autoimmune reactions against this class of self-antigens. Similarly, if a CD8^+^ T cell is found to cross-react with self-peptides while still back in the thymus, this self-reactive CD8^+^ T cell will be converted into a clone of CD8^+^ T_REG_ cells, involved in immunosuppression.

However, for a partially activated CD4^+^ T cell that has no self-reactivity while back in the thymus, this T cell may be transformed into a special subset of CD4^+^ helper T cells if it later encounters the same antigen again in the periphery. It is speculated that these helper T cells may play an important role in mediating the body’s anticancer immunity or autoimmunity.

The proposed hypotheses are of value in offering a different angel in looking at some of the immensely complex functions of the immune system, including the selective antigen presentation, the formation of CD8^+^ cytotoxic T cells, and the formation of T_REG_ cells. As discussed in the paper, there are some experimental (mostly indirect and circumstantial) observations which offer partial, tangible support for the proposed hypotheses. It is hoped that the proposed hypotheses will help promote the thinking, debate and experimental testing of the molecular mechanisms underlying the intriguing and immensely-complex process of selective antigen presentation.

## Data Availability

No new data were created or analyzed in this study. Data sharing is not applicable to this article.

## Abbreviations

The following abbreviations are used in this manuscript:

MHC-I	major histocompatibility complex class I molecule
MHC-II	major histocompatibility complex class II molecule
TCR	T cell receptor
BCR	B cell receptor
iTCR^I^	intracellular version of the TCR protein functionally coupled with MHC-I molecule
iTCR^II^	intracellular version of the TCR protein functionally coupled with MHC-II molecule
APC	antigen-presenting cell
DC	dendritic cell
T_REG_ cell	regulatory T cell
tT_REG_ cell	thymic T_REG_ cell
pT_REG_ cell	peripheral T_REG_ cell
CTL	cytotoxic T lymphocyte
CTL-P	cytotoxic T lymphocyte precursor
EV	extracellular vesicle
IFN	interferon
PAMP	pathogen-associated molecular pattern
PRR	pattern recognition receptor
PLC	peptide-loading complex
RER	rough endoplasmic reticulum
mTEC	medullary thymic epithelial cell
